# Ten-Year Evaluation of Thermal Comfort in Operating Rooms

**DOI:** 10.3390/healthcare10020307

**Published:** 2022-02-05

**Authors:** Giovanna Deiana, Antonella Arghittu, Marco Dettori, Maria Grazia Deriu, Alessandra Palmieri, Antonio Azara, Paolo Castiglia, Maria Dolores Masia

**Affiliations:** 1 Department of Biomedical Sciences, University of Sassari, 07100 Sassari, Italy; giovanna.deiana90@gmail.com (G.D.); arghittu.antonella@gmail.com (A.A.); 2 University Hospital of Sassari, 07100 Sassari, Italy; mariagrazia.deriu@aousassari.it (M.G.D.); luca@uniss.it (A.P.); azara@uniss.it (A.A.); castigli@uniss.it (P.C.); 3 Department of Medical, Surgical and Experimental Sciences, University of Sassari, 07100 Sassari, Italy; mdmasia@uniss.it

**Keywords:** microclimate, thermal comfort, operating rooms

## Abstract

The microclimate is a particularly important environmental aspect in operating rooms (ORs), where more than in other hospital environments, it is extremely important, and at the same time extremely difficult, to reconcile the needs of different types of occupants (patients and operators). Moreover, unsuitable microclimatic conditions may affect the onset of infection. The present study aimed to analyze the periodic monitoring of the microclimatic conditions carried out in ORs over 10 years, to verify the adequacy of the thermal comfort conditions for all occupants. The evaluation of thermal comfort was carried out using the Fanger indices and the standards required by current legislation and specific guidelines. Non-compliant values for at least one parameter were found in 98.8% of the examinations performed in the ORs. A condition of thermal discomfort was calculated for 3.6% of healthcare professionals and 98.3% of patients. The monitoring of microclimatic conditions is particularly important in the OR as an indicator of inadequate functioning of the air conditioning system, which might affect the thermal comfort of all occupants and lead to microbial contamination of the room.

## 1. Introduction

The quality of living and working environments, according to the American Society of Heating, Refrigerating and Air-Conditioning Engineers (ASHRAE), is influenced by the microclimate, i.e., a set of environmental parameters, such as temperature, humidity, airspeed and radiant heat, which condition the heat exchange between people and the environment and contribute to attaining thermal comfort [1,2]. Thermal comfort is the sensation of a human body exchanging heat with the surrounding environment without forcing the thermoregulatory mechanisms it is equipped with. On the contrary, a condition of thermal discomfort can arise due to the influence of unfavorable microclimatic characteristics. If microclimatic characteristics remain unfavorable or worsen, real thermal stress can occur, causing a set of pathophysiological effects when the thermohygrometric conditions exceed the body’s ability to thermoregulate [3,4].

The microclimate is a particularly important environmental aspect in the hospital setting, not only for its occupants who—being affected by health problems—are more sensitive than the general population to environmental risk factors but also because—being a workplace—it must guarantee adequate and effective protection of the operators [5,6,7]. Moreover, in the hospital environment, the microclimatic characteristics are especially significant in operating rooms (ORs), where more so than in other hospital environments, it is extremely important, and at the same time extremely difficult, to reconcile the needs of different types of occupants, both relating to the patients’ homeothermia and various components for the operating team, whose performances can be significantly affected by any thermal discomfort, with possible negative consequences for the health of patients [8,9,10].

Furthermore, the level of environmental microbial contamination in the OR must be taken into consideration among the various factors that can determine the onset of surgical site infections (SSIs), as unsuitable microclimatic conditions can affect the microbiological characteristics of the air, favoring the formation of microbial aerosols and the deposition of contaminating particles, and therefore, the onset of infections [11,12,13]. In this regard, SSIs are the second most frequent cause of healthcare-associated infections (HAIs) in Europe and the United States and the most common type of HAIs in low- and middle-income countries. Overall, SSIs are linked to one-third of post-operative deaths and represent 8% of all deaths caused by HAIs [14,15].

Based on these considerations, the present study aimed to analyze the periodic monitoring of the microclimatic conditions of ORs of the University Hospital of Sassari over 10 years, to verify the adequacy of the thermal comfort conditions of all occupants. The research also aimed to underline the importance of proactive environmental surveillance as a useful strategy for preventing nosocomial infections [16,17,18].

## 2. Materials and Methods

### 2.1. Setting

The study, covering the period from January 2010 to December 2019, involved testing in 18 ORs in the University Hospital of Sassari, the main hospital in Sardinia in terms of the number and heterogeneity of its technological and professional resources (861 beds and 3044 employees), with a catchment area of 600,000 inhabitants. The rooms, covering various specialties, are all equipped with turbulent flow ventilation and air conditioning systems. Sampling was performed by members of the Laboratory of the Hygiene and Hospital Infection Control Operative Unit at the University Hospital, according to the methods reported below.

### 2.2. Sampling

Assessment of the microclimatic conditions inside the ORs was carried out using a data acquisition instrument (model: BABUC version 5.07 LSI, data logger satisfying ISO 9001 regulations), consisting of a multi-acquisition device mounted on a mobile and adjustable stand, connected to a group of sensors: a psychrometer with forced ventilation, along with a distilled water tank to detect the dry bulb temperature and relative humidity; a globe thermometer to determine the mean radiant temperature; a hot wire anemometer to measure the airspeed.

The instrument was placed near the operating field, in a point not subject to rapid air displacements, at least 1 m from the wall and 1.5 m from the floor [19]. The measurements were carried out with the air conditioning system in operation.

According to the Guidelines on Occupational Safety and Hygiene Standards in the Operating Room outlined by the ISPESL (Istituto Superiore per la Prevenzione e la Sicurezza del Lavoro), measurements were taken both with the OR empty (“at rest”), to evaluate the functioning of the heating, ventilation and air-conditioning (HVAC) systems, and throughout the surgery (“in operation”), to determine both the functioning of the HVAC systems and the thermal comfort of staff and patients [19].

The evaluation of thermal comfort was carried out using Fanger’s indices: PMV (predicted mean vote) and PPD (predicted percentage dissatisfied), according to ISO 7730 standard values [20]. Thus, using “InfoGap” software supplied to the BABUC, the climatic data collected were subsequently processed by selecting from a specific menu both the energy expenditure of the people present in the operating room (operators and patients) and the thermal resistance provided by the clothing worn, based on ISO 9920 standard values. The metabolic energy heat was measured in *met* (1 *met* = 58 W/m^2^), while the thermal resistance of clothing was measured in *clo*, an abbreviation of the term clothing (1 *clo* = 0.155 °C/W). For the occupants of the operating room, the following values were selected: Operator = *met* 1.599 and *clo* 0.6; Patient = *met* 0.8 and *clo* 0.31.

Information relating to the thermal comfort of the patient was not detected in the cardiac surgery rooms as their specific surgical activity required particular temperature characteristics during the operation, and for children, as the instrument was calibrated to calculate the average thermal comfort of an adult man.

Results were interpreted according to the standards required by current legislation and specific guidelines relating to the climatic conditions in ORs and indices of thermal comfort, and based on the values of PMV and PPD indicated as acceptable by ASHRAE [1,19,21,22]:

- Air temperature: from 20 °C to 24 °C;
- Relative humidity: from 40% to 60%;
- Mean radiant temperature: must be within +/−2 °C of the air temperature;
- Airspeed: from 0.05 m/s to 0.15 m/s;
- PMV: ±0.5 (recommended value); ±0.85 (acceptable value);
- PPD: ≤10% (recommended value); ≤20% (acceptable value).

### 2.3. Statistical Analysis

Data were entered on Excel (Microsoft Office, Microsoft Corporation, Redmond, WA, USA) and analyzed using STATA software, version 16 (StatCorp., Austin, TX, USA). Qualitative variables were summarized as absolute and relative (percentages) frequencies, whereas quantitative variables were summarized with position measures and the variability of values, depending on their parametric distribution. The one-sample test of proportion was performed to obtain 95% confidence intervals.

## 3. Results

In the period 2010–2019, a total of 169 investigations were carried out. Of these, for organizational reasons, 138 (81.7%) were carried out both in empty rooms and during surgery, 29 (17.1%) only at rest and 2 (1.2%) only in operational conditions, for a total of 307 samples (167 at rest and 140 in operation). Depending on technical and organizational reasons (the room’s specialty, maintenance interventions, renovation of the air conditioning system, etc.), the number of investigations per room varied year-on-year, as reported in Table 1.

**Figure 1:**
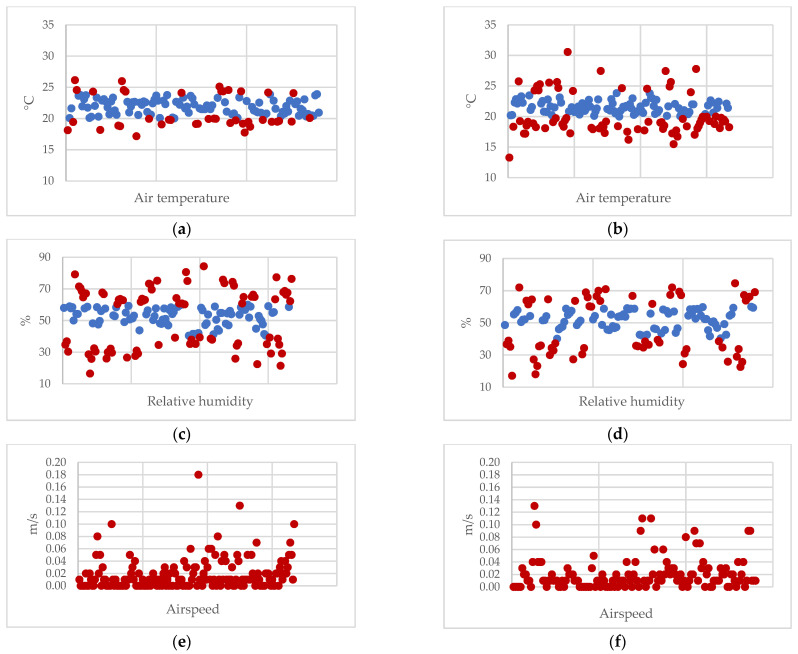
Distribution of the values of air temperature (**a**,**b**), relative humidity (**c**,**d**), and airspeed (**e**,**f**) at rest (**a**,**c**,**e**) and in operation (**b**,**d**,**f**) in comparison with the reference values. Blue dot = compliant values; Red dot = non-compliant values.

Non-compliant values for at least one parameter were found in 98.8% (167/169) of the investigations performed in the ORs; among these, 79% (132/167) were both at rest and in the operation, 18% (30/167) were only when the room was empty and in 3% (5/167) were only during surgery. Ninety-seven percent (162/167) of the samplings carried out at rest and 97.1% (136/140) of those in the operation were found to be outside the norm. The airspeed was the parameter most often outside of the standard range (88.6% of the samples; 87.4% at rest and 90% in the operation), while the radiant temperature was almost always compliant (92.5% of the samples; 88.1% at rest and 97.9% in the operation). To a lesser extent, non-standard values were found for the relative humidity (45.6% of the samples; 48.5% at rest and 42.1% in the operation) and air temperature (34.9% of the samples; 43.1% at rest and 25% in the operation). Only two examinations met the parameters, in 2016 (OR 3) and 2017 (OR 6). Figure 1 represents the distribution of the values of the parameters in comparison with the reference values.

**Figure 2:**
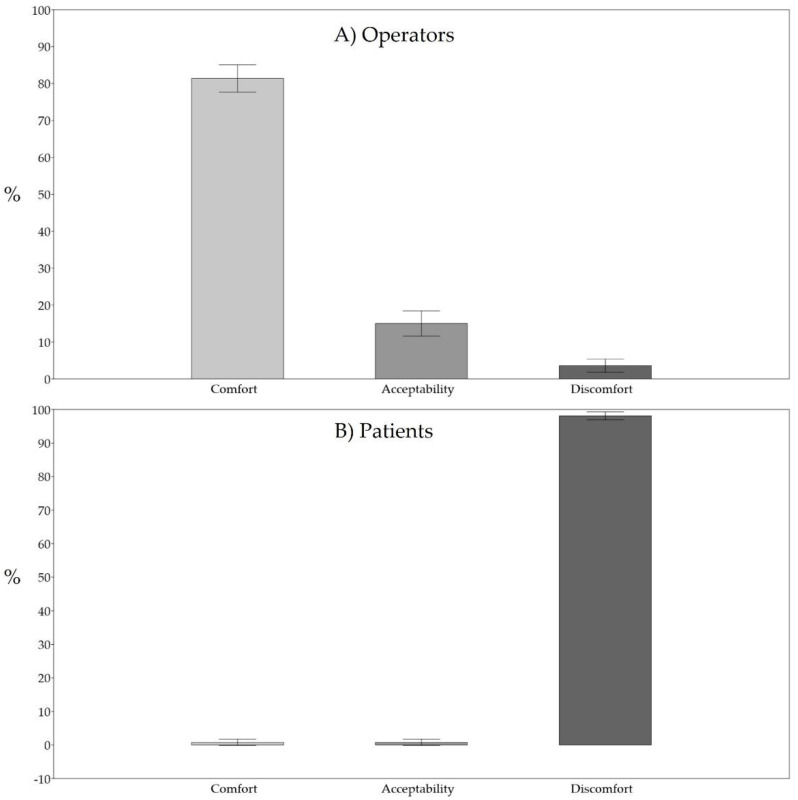
Values and 95% confidence intervals of thermal comfort, acceptability and discomfort for operators (**A**) and patients (**B**).

The Fanger indices developed for healthcare professionals showed thermal discomfort in 3.6% of cases, while 98.3% of patients experienced discomfort (Table 2 and Figure 2).

In particular, concerning the thermal discomfort of operators, as stated by ASHRAE [1], Fanger’s indices showed slightly cool and slightly warm conditions in 80% and 20% of unsatisfactory cases, respectively. On the other hand, concerning patients, their thermal discomfort was caused by conditions that were slightly cool, cool and cold in 5.1%, 5.1% and 89.8% of cases, respectively.

## 4. Discussion

The experiment we that conducted highlighted, in almost all the ORs examined, both at rest and in operating conditions, the non-conformity of one or more microclimatic parameters with the standards required by current legislation and specific guidelines. This demonstrates the inadequacy of the surgical interventions carried out over the years and underscores the need for revision or adaptation of the air conditioning systems, as well as improved management of the OR, to control various structural, organizational and functional variables capable of influencing the microclimate. This is fundamental as unsuitable microclimatic conditions can influence the levels of environmental microbial contamination, and therefore, the onset of SSI.

Our results confirm an issue highlighted by previous studies [23,24] and differ from those that presented compliant values for microclimatic monitoring in as many as 75% of cases. Yet, it is difficult to conclude in this sense as there are very few studies in the literature that evaluate the microclimatic characteristics in the OR [25].

Satisfaction with the thermal environment in the healthcare setting is based on a complex and subjective reaction to certain variables. In ORs, it is fundamental, and at the same time extremely difficult, to reconcile the needs of different occupants: the patient, under anesthesia and practically naked, needs quite a high temperature and humidity to avoid perfrigeration and minimize dehydration of the exposed tissues; the operating team, on the other hand, favor a relatively cool, well-ventilated and dry environment as they have to sustain high performance for a prolonged period. Furthermore, individual physiological characteristics, and the different activities performed, might affect the thermal comfort levels of healthcare personnel [26,27,28,29,30,31].

We found that a condition of thermal comfort, or at least, of thermal acceptability for the operators is almost always guaranteed (96.4%). Thermal discomfort of the operators mostly arose when the operating team wanted or required certain climatic conditions to suit a particular type of intervention. Conversely, the climatic characteristics observed when considering the energy expenditure of the patient and the thermal impedance offered by their clothing worn revealed that they frequently experienced cold stress, which was sometimes very severe, as evidenced by PMV values beyond -3 on the ASHRAE scale.

In a previous study, the authors observed that for the various combinations of microclimatic parameters included in the regulatory standards provided, the Fanger indices consistently highlight the existence of a thermal gap between operators and patients [23]. The different conditions of thermal comfort/discomfort of operators and patients with the same microclimatic conditions, also found in other investigations [24], confirm the importance of the individual variables of metabolic expenditure and thermal resistance of clothing. Surgical clothing is noted to be designed taking into account certain thermal comfort components, such as thermal resistance, air permeability, water vapor resistance and flexibility [32,33,34].

The studies conducted on this topic have focused above all on the thermohygrometric comfort of healthcare workers. Nevertheless, the anesthesiology community has focused on the risks for the patient caused by perioperative changes in normothermia, highlighting the need to also direct studies toward identifying microclimatic characteristics suitable to guarantee the thermal comfort of the patient in the OR. In this regard, the environmental temperature is recognized, especially in extreme ages, as the critical factor for the development of perioperative hypothermia, which is the effect on thermoregulation that most frequently follows anesthesia. While a mild degree of controlled hypothermia can be useful in some surgical specialties, such as neurosurgery, cardiac surgery and vascular surgery, or when you want to protect tissues from ischemic damage, accidental hypothermia, even mild, is often harmful [23,35].

In particular, in the intraoperative period, hypothermia is associated with a decrease in the clearance of anesthetic drugs and curariums, changes to coagulation and an increase in hematocrit, while, in the postoperative period, hypothermia promotes desaturation and ischemic accidents of the myocardium. Hypothermic patients also present a higher incidence of infection of the surgical wound, attributable to vasoconstriction, due to the hyperincretion of catecholamines in response to cold stress, and to the decrease in the phagocytic activity of leukocytes as a result of the reduced oxygen supply at the wound site. Finally, the reduction in core temperature leads to a lengthening of the re-awakening times from anesthesia and greater degrees of postoperative pain [7,36]. This justifies the increasingly frequent use by anesthesiologists, for the prevention of perioperative hypothermia, of methods of heating anesthetic gases and infusion solutions, in addition to the adoption of active external heating systems for the patient himself.

Monitoring the microclimate in the OR is even more important considering the known correlation between infections spread via the air and the microclimate. The different microclimatic elements condition the formation of microbial aerosols as the turbulence of the air modifies the sedimentation forces of the aerosols and the heat activates the convective movement of the air, favoring ascending currents that carry the suspended particles. Likewise, the humidity of the environment influences the sedimentation of the aerosols by modifying the evaporation speed of the droplets that remain dispersed for a long time in the environment, with the ability to move and spread over considerable distances [10,37,38,39].

We must note that certain limitations affect the reliability of the results of the present study. Firstly, although the system adopted identifies and considers various individuals (patients, operators with different roles), the data processed refer to the average man, as happens for many medical data (e.g., drug dosages), not considering that the individual variables that affect thermal comfort may be affected by differences in gender and age. Nonetheless, the survey methodology adopted, which proved to be simple, practical and effective, allowed us to calculate, under the same climatic conditions, the thermal comfort/discomfort for all types of occupants. A second limitation of this work is the few comparisons presented with other studies conducted at the national and international levels as there are very few articles on the subject in the literature [7]. When reconsidered, this aspect also represents a strength of our work as we have highlighted something that is rarely mentioned, i.e., the importance of proactive environmental surveillance of the microclimate, and consequently, of thermal comfort in the operating room.

## 5. Conclusions

When considering the activities carried out in the OR, climatic comfort is particularly important not only because negative thermal sensations can produce a state of discomfort capable of compromising the quality of the operating team’s performance but also for the maintenance of normothermic conditions in the patient. Therefore, microclimatic characteristics must be monitored, with a predefined periodicity, to verify the healthiness of the air and as an indicator of sentinel events. Indeed, altered microclimatic conditions might be a possible indicator of inadequate functioning of the air conditioning system, which in turn, could lead to microbial contamination of the room.

In future research, we advise associating instrumental evaluations with subjective evaluations of the comfort gained through specific questionnaires administered during monitoring, thus arriving at a more realistic evaluation of the thermal comfort, which is essential to have when trying to satisfy the needs of all occupants [31].

## Figures and Tables

**Table 1:** Distribution of examinations per year and condition.

Year	No. ORs Monitored	Examinations	AR Samples	OP Samples	AR + OP	AR Only	OP Only
2010	15	22	21	19	18	3	1
2011	9	10	10	6	6	4	0
2012	15	15	15	13	13	2	0
2013	18	27	27	21	21	6	0
2014	18	19	19	14	14	5	0
2015	15	15	15	12	12	3	0
2016	18	21	21	17	17	4	0
2017	14	15	14	13	12	2	1
2018	14	14	14	14	14	0	0
2019	11	11	11	11	11	0	0
Total		169	167	140	138	29	2

AR = at rest; OP = in operation.

**Table 2:** Values of thermal comfort, acceptability and discomfort for operators and patients.

	Operators	Patients
Comfort	114/140 (81.4%)	1/118 (0.8%)
Acceptability	21/140 (15.0%)	1/118 (0.8%)
Discomfort	5/140 (3.6%)	116/118 (98.3%)

## Data Availability

The data presented in this study are available on reasonable request from the corresponding author.
